# Population decline: where demography, social science, and biology intersect

**DOI:** 10.1530/REP-24-0070

**Published:** 2024-05-31

**Authors:** Robert John Aitken

**Affiliations:** 1 Priority Research Centre for Reproductive Science, Discipline of Biological Sciences, School of Environmental and Life Sciences, College of Engineering Science and Environment, University of Newcastle, Callaghan, New South Wales, Australia; 2 Hunter Medical Research Institute, New Lambton Heights, New South Wales, Australia

## Abstract

**In brief:**

Over the past half century, the world has witnessed an unprecedented decline in human fertility rates. This analysis reviews the various socioeconomic, cultural, and biological factors involved in driving this change and considers whether low fertility rates are a temporary or permanent feature of our future demographic profile.

**Abstract:**

Since the early 1960s, the world has witnessed the spectacular collapse of human fertility. As a result of this phenomenon, several countries are already seeing their population numbers fall and more will follow in the coming decades. The causes of this fertility decline involve a complex interplay of socio-economic, environmental, and biological factors that have converged to constrain fertility in posterity’s wake. Since large numbers of offspring are no longer needed to compensate for high infant mortality in contemporary society, couples have opted to have small families in a quality-over-quantity investment in their progeny’s future. Simultaneously, increases in female education, the enhanced participation of women in the paid workforce, and a resultant delay in childbearing has placed limits on achievable family size. Progressive urbanization, the improved availability of contraceptives, and the socio-economic pressures experienced by young adults in ageing societies are also contributing to fertility’s demise. These factors, together with the individualism that pervades modern society and the increasing social acceptability of voluntary childlessness, have firmly established a low fertility ethos in most post-transition countries. Since none of these forces are about to relent, it looks as if extremely low fertility might be with us for some time to come. This may have long-term consequences. The lack of selection pressure on high fertility genotypes, the ability of ART to retain poor fertility genotypes within the population, and sustained exposure to reproductive toxicants in modern industrialized environments may all contrive to leave a permanent mark on the fecundity of our species.

## Introduction

**Figure 1:**
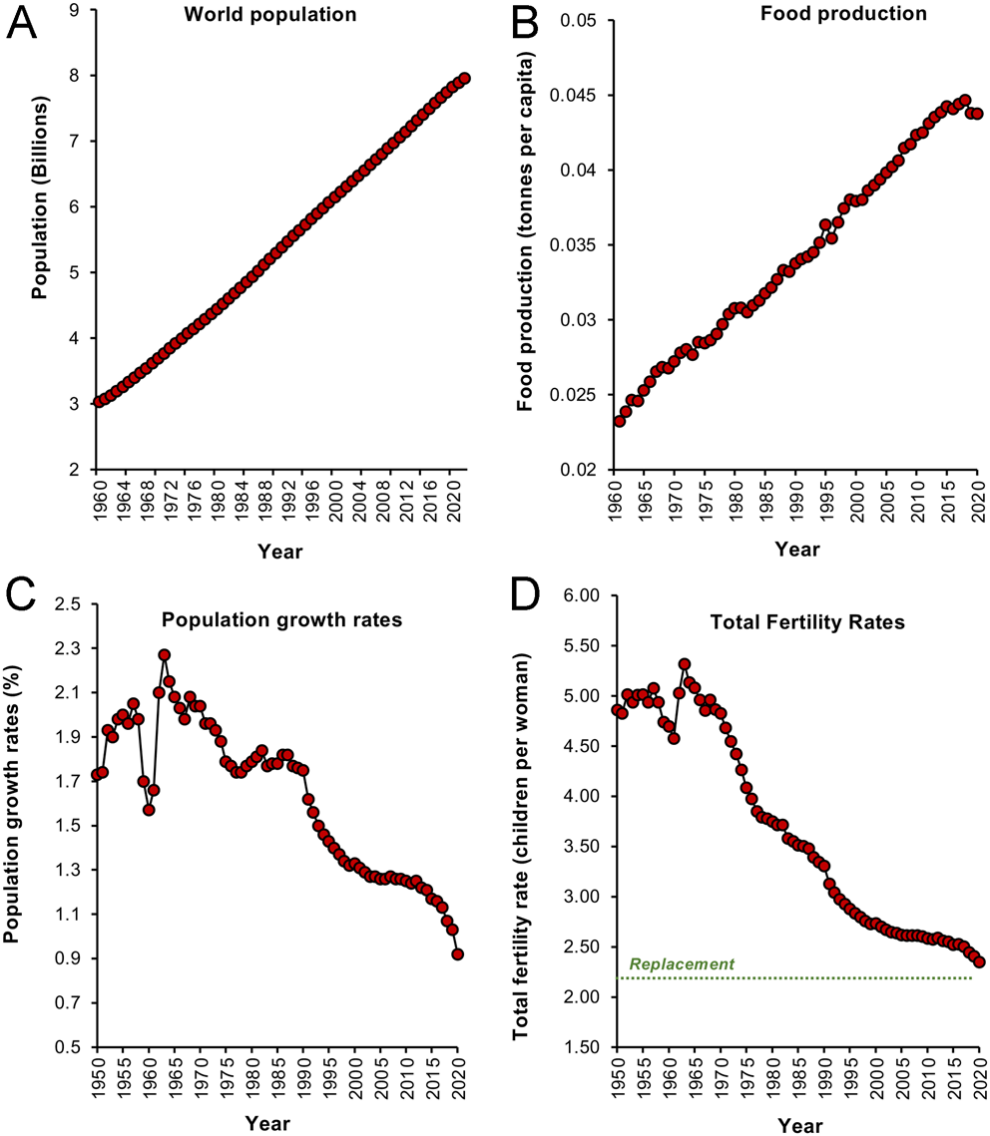
Changes in human population dynamics since 1960. (A) Linear growth of the World’s population from 1960 to 2022. (B) Food production per capita over the same period of time. (C) Population growth rate. (D) Total fertility rates; replacement level fertility is 2.1 children per woman. Sources: (A) World Bank Open Data (https://data.worldbank.org/indicator/SP.POP.TOTL); (B) Our World in Data (https://ourworldindata.org/agricultural-production); (C) Our World in Data (https://ourworldindata.org/grapher/population-growth-rates); (D) United Nations World Population Prospects (https://population.un.org/wpp/Download/Standard/MostUsed/).

Publication of *The Population Bomb* by Paul Ehrlich in 1968 captured growing concerns about the rate of world population growth and energized a generation of reproductive biologists to undertake research on the regulation of human fertility (Ehrlich 1968). In this book, Ehrlich proposed that the rapid growth of the world population, coupled with high rates of per capita consumption in the most economically advantaged societies, would put relentless pressure on the resources, particularly food, needed to sustain our species. Indeed, in a 1971 speech, Ehrlich predicted that ‘By the year 2000 the United Kingdom will be simply a small group of impoverished islands, inhabited by some 70 million hungry people’. In the intervening half century, global population growth has indeed continued apace and will peak at around 10 billion before the end of century (United Nations 2024, Fig. 1A). However, the Malthusian disaster foreseen by Ehrlich has not materialized, largely because we have witnessed, in parallel, a technical revolution in agriculture so that our capacity to generate food has actually increased in concert with the growth of our population in a very non-Malthusian paradox (Fig. 1B). Freed from the chains of starvation and bolstered by the miracles of modern medicine that have both extended lifespan and reduced infant mortality rates, our species has experienced a frighteningly rapid population expansion over the past century.

In the early 1970s, most opinion leaders in reproductive science were convinced that part of the solution to world population growth would be to develop improved forms of fertility control. Luminaries such as Roger Short, Malcolm Potts, Egon Diczfalusy, Richard Ivell, Wayne Bardin, and Mike Harper triggered a surge of research aimed at securing a deeper understanding of the fundamental mechanisms controlling human reproduction with the ultimate aim of using this knowledge to develop new approaches to contraception (Aitken *et al.* 2008). Institutes like the MRC Reproductive Biology Unit in Edinburgh, the Population Council’s Center for Biomedical Research in New York, and the Institute for Hormone and Fertility Research in Hamburg were all established with this in mind. Major pharmaceutical companies like Organon and Schering AG actively funded this area, while the World Health Organisation’s Human Reproduction Unit co-ordinated and resourced contraceptive research through its various Task Forces. Some notable successes emerged from these programmes, including the progesterone antagonist RU486 (Nieman *et al.* 1987) and a plethora of systems (vaginal rings, implants, subcutaneous depots, etc.) for presenting steroids to the endocrine system. In the wake of this research, undertaking the rate of human population growth has, indeed, started to slow (Aitken 2022*a*). However, improvements in contraceptive technology were not primarily responsible for this change; that responsibility falls to a complex collection of factors involving our culture, our society, and our fundamental reproductive biology.

## The rise and fall of global fertility

Our species gives birth to extremely immature, neotenous young that require many years of post-natal care to develop the point that they can contribute to the society into which they were born. In order to optimize the chances of offspring survival, our fecundity (our fundamental capacity to reproduce as reflected by the probability of achieving conception within a given menstrual cycle) is relatively low compared with other mammalian species, as part of an evolutionary quality–quantity trade-off (Kaplan 1996). Thus, in our species, fecundity is limited to 0.2−0.3 (i.e. a 20–30% chance of becoming pregnant in a given menstrual cycle) compared with a figure of 0.9 for certain strains of laboratory mouse (Bongaarts 1975, Silver 1995, Zinaman *et al.* 1996, Joffe 2010). The human fecundity setting may seem relatively low; however, it has been sufficient to allow Neolithic women to produce an average of 4–6 children during their reproductive lifespan (Wilson 2001, Jones & Bird 2014, Page *et al.* 2016, Page & French 2020). Moreover, since at least half of these children would die before they reached sexual maturity (Volk & Atkinson 2013), the seesaw of life and death for our species was more or less balanced, and our population remained at less than 0.5 billion throughout most of history. Then, in the late 18th century, everything changed. Triggered in part by the affluence, technical know-how and improved healthcare associated with the First Industrial Revolution, and in part by the philosophical awakening associated with the Enlightenment, the human population commenced its unprecedented global expansion. These high growth rates were only temporary, however. We are now on the cusp of a sudden unexpected reversal in population numbers, the first inklings of which were signalled by a sudden change that occurred in 1964, just before Ehrlich published his book.

**Figure 2:**
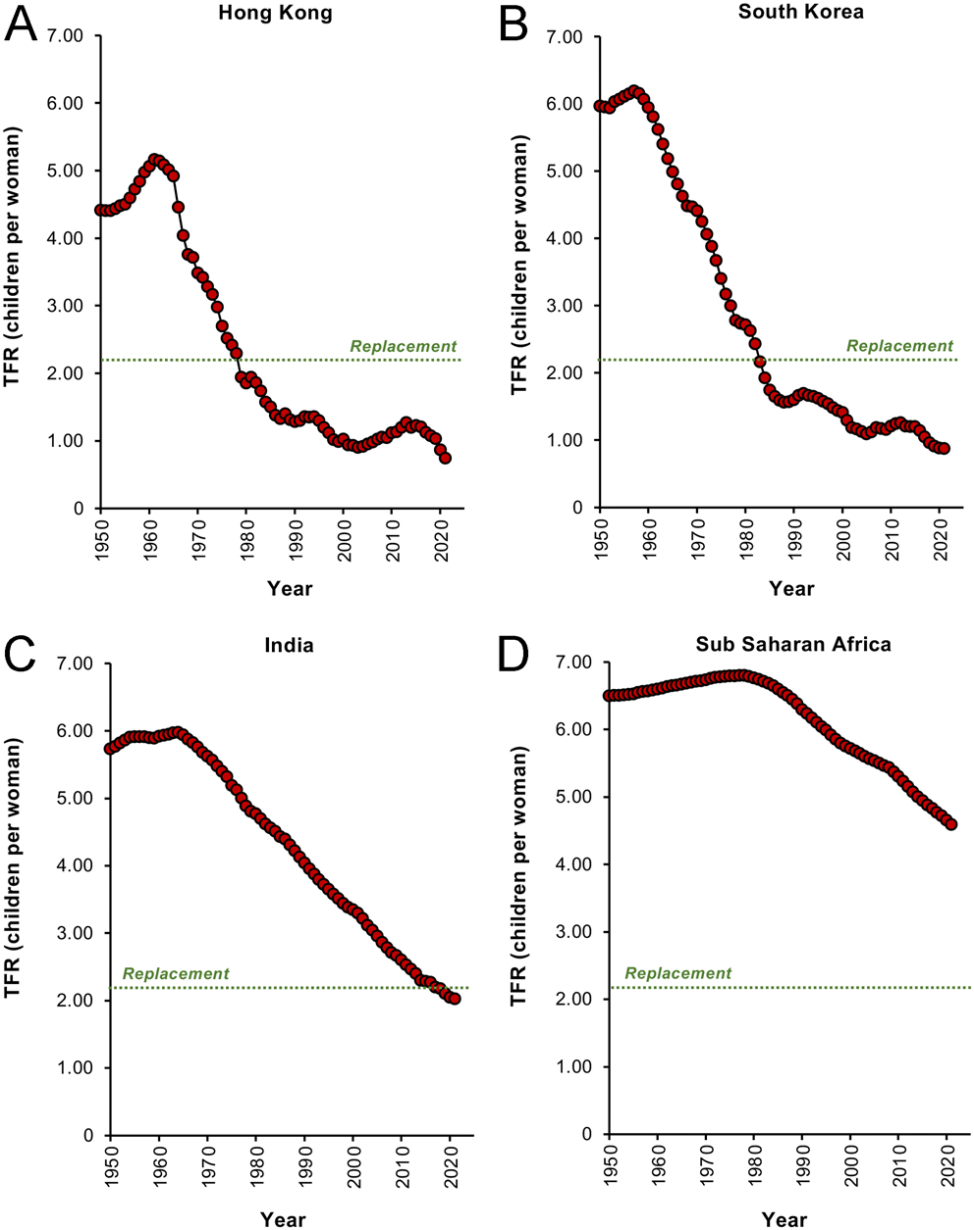
Changes in total fertility rates (TFRs) in individual countries. (A) Hong Kong, (B) South Korea, (C) India, (D) sub-Saharan Africa. Replacement level fertility is 2.1 children per woman. Source: World Bank Open Data (https://data.worldbank.org/indicator/SP.DYN.TFRT.IN)

Examination of UN population data since 1950 (Fig. 1C) reveals how the rate of population growth suddenly slowed in 1964 before commencing a full-scale retreat that continues to this very day (Fig. 1C). This reduction in growth rate has been associated with a parallel fall in global total fertility rates (TFR refers to the average number of children a woman will produce during her reproductive lifetime assuming current age-specific fertility rates) across the globe. Worldwide, TFR has been in dramatic decline since the mid-1960s, and we are now perilously close to the replacement threshold of 2.1 (the number of children a women must have to sustain the population, 2 to replace the parents, and 0.1 to compensate for an inability to procreate because of death or infertility). Indeed, if we look at individual countries, there are already several advanced economies that are exhibiting TFRs well below this replacement level and have been for some time. In the vanguard of this trend are the Tiger economies of East Asia such as Singapore, Taiwan, Hong Kong (Fig. 2A), and South Korea (Fig. 2B), as well as countries in the same geographical region such as China and Japan. Extremely populous nations such as India have recently seen their TFR decline below 2.1 (Fig. 2C) and even the highly fertile nations of sub-Saharan Africa have seen their fertility rates fall over the past 40 years (Fig. 2D). Vollset *et al.* (2020) have predicted that by 2050, 151 countries will have a TFR lower than the replacement level and by 2100 that number will have grown to 183.

Once the replacement TFR threshold has been crossed, we might expect to see a sudden decline in overall population numbers. However, this does not occur immediately for two major reasons: (i) the improved healthcare provided by modern industrialized societies have generated an increase in life span, so total population numbers turn over less rapidly; and (ii) the impact of *population momentum*. The latter essentially defines the means by which populations can continue to grow even though their TFR has fallen to below replacement level. It is a buffering mechanism whereby populations can harvest the benefits of their previous fecundity as a result of the continuing entry of young women into the reproductive age cohort. In India, for example, TFRs have already fallen below the replacement value of 2.1 but the population will continue to grow for decades to come. This is due to the large number of young women (the Indian population contains 173 million girls under 14 years of age) who will be entering the reproductive cohort over that period of time and contributing to sustained population growth even though national fertility rates are at an all-time low. Eventually, however, this momentum will dissipate, and the population will contract.

**Figure 3:**
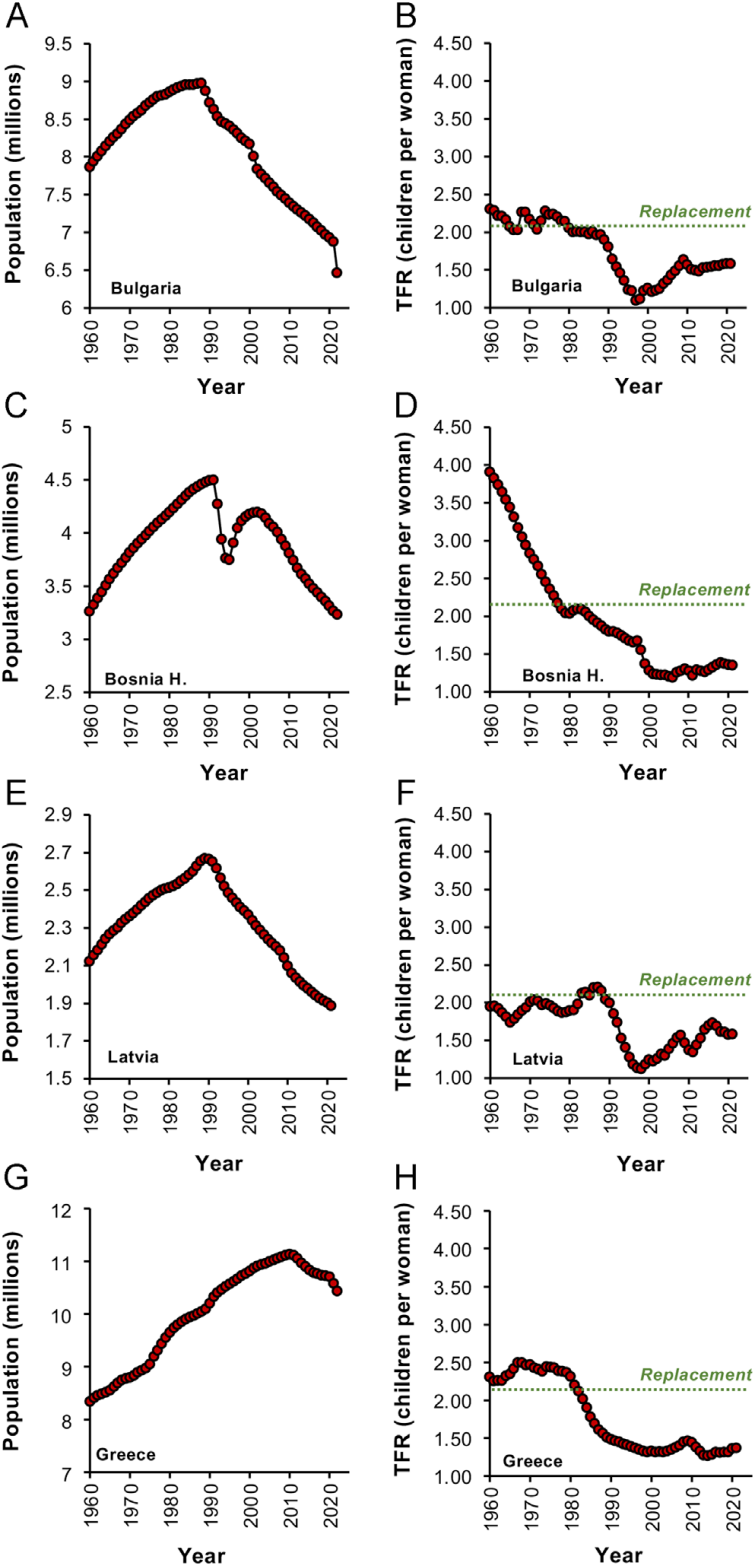
Changes in population numbers in some of the most vulnerable countries in the world. Left hand panels show the rise and fall of population numbers for each country since 1960 while the right-hand panels show the corresponding TFR values, which have been consistently below replacement level. (A) and (B), Bulgaria; (C) and (D) Bosnia Herzegovina; (E) and (F) Latvia; (G) and (H) Greece. Source: World Bank Open data (https://data.worldbank.org/indicator/SP.DYN.TFRT.IN and https://data.worldbank.org/indicator/SP.POP.TOTL).

There are some countries where this has already happened. Publicly available databases such as World Bank Open Data (2024) indicate that the Chinese population is already starting to decline, and Japan is losing around 0.5 million inhabitants a year. Particularly badly affected are a group of ex-communist states in Central Europe (e.g. Belarus, Georgia, Bulgaria, Hungary, Ukraine, Moldova), several countries in the Balkans (Albania, Serbia, Bosnia Herzegovina, Croatia), and the Baltic States (Estonia, Latvia, Lithuania), examples of which are given in Fig. 3A–F. A large collection of countries also exist which have experienced extremely low TFR values for a long period of time and are now on the brink of population decline once the buffering impacts of longevity and population momentum have been exhausted. Included in this group of countries are the Tiger economies, Japan, Poland, and Greece (Fig. 3G and H). The social, economic, and geopolitical consequences of such shrinking, ageing populations are immense and beg three key questions, ‘Why is this decline in human fertility occurring?’ ‘What are the long-term ramifications for our species ?’ and ‘Can population numbers be controlled?’ The answers to these questions can only be reached when we have achieved a more integrated and coherent interdisciplinary understanding of this complex area (Balbo *et al.* 2013).

### Why is this decline in human fertility occurring?

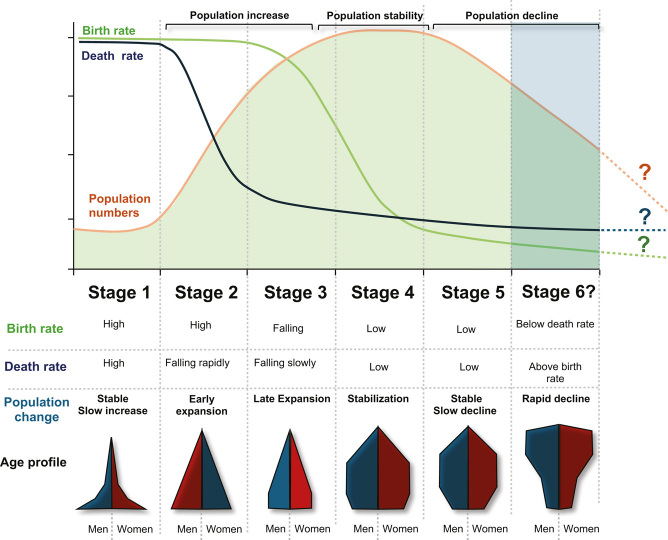

THE DEMOGRAPHIC TRANSITION. In Stage 1 of this process birth and death rates are both relatively high and population size remains relatively stable. This Stage represents most of human history, when life was nasty, brutish and short and human populations were held in check by the ravages of pestilence, famine and war. Stage 2 of the Demographic Transition is associated with the sudden expansion of population numbers as high birth rates became coupled with reductions in mortality, particularly infant mortality, in the wake of increased prosperity and knowledge. As societies enter Stage 3 of the Demographic transition birth rates suddenly start to fall even though the resources are available to sustain a growing population. This paradoxical non-Mathusian situation, covers most developing countries today. Stage 4 of the Demographic Transition covers a time of stabilization when birth and death rates are low, the economy is strong, education levels are high, and an increasing number of women are entering the paid workforce. During this stage of demographic evolution, the TFR vales are hovering around the replacement threshold. The phase ultimately gives way to Stage 5 of the Demographic Transition when TFRs are well below replacement and the population is rapidly aging; this is the Stage that characterizes most advanced economies at the present time. Stage 6 of the demographic transition represents unchartered waters. If current trends continue, sustained low fertility and population decline will be inevitable.

The demographic transition describes the way in which human population growth changes as societies become more socio-economically advanced and resources are increasing. During stages 4 and 5 of this process, death and birth rates are low and the tide of population growth is on the turn (Box 1). The nature of the forces contributing to fertility decline during the late stages of transition have been much contested and are often coloured by the discipline lens through which this phenomenon is viewed. In this context, reductions in TFR should be recognized as a global phenomenon that cut across major differences between nations and communities in terms of religion, ethnicity, political persuasion, or culture. In searching for the primary forces driving fertility change, the factors that differentiate societies are not as important as the elements that are shared. Overall, the global decline in TFR appears to be due to the convergence of several factors rather than any single cause operating in isolation. Thus, declines in infant mortality as well as increases in resource availability, education, contraception, urbanization, and developmental idealism are all prominent in this process and, as we shall discuss, closely inter-related (Thornton 2001, Angeles 2010, Bloom *et al.* 2011, Murtin 2013, Aitken 2022*a*).

### Infant mortality

**Figure 4:**
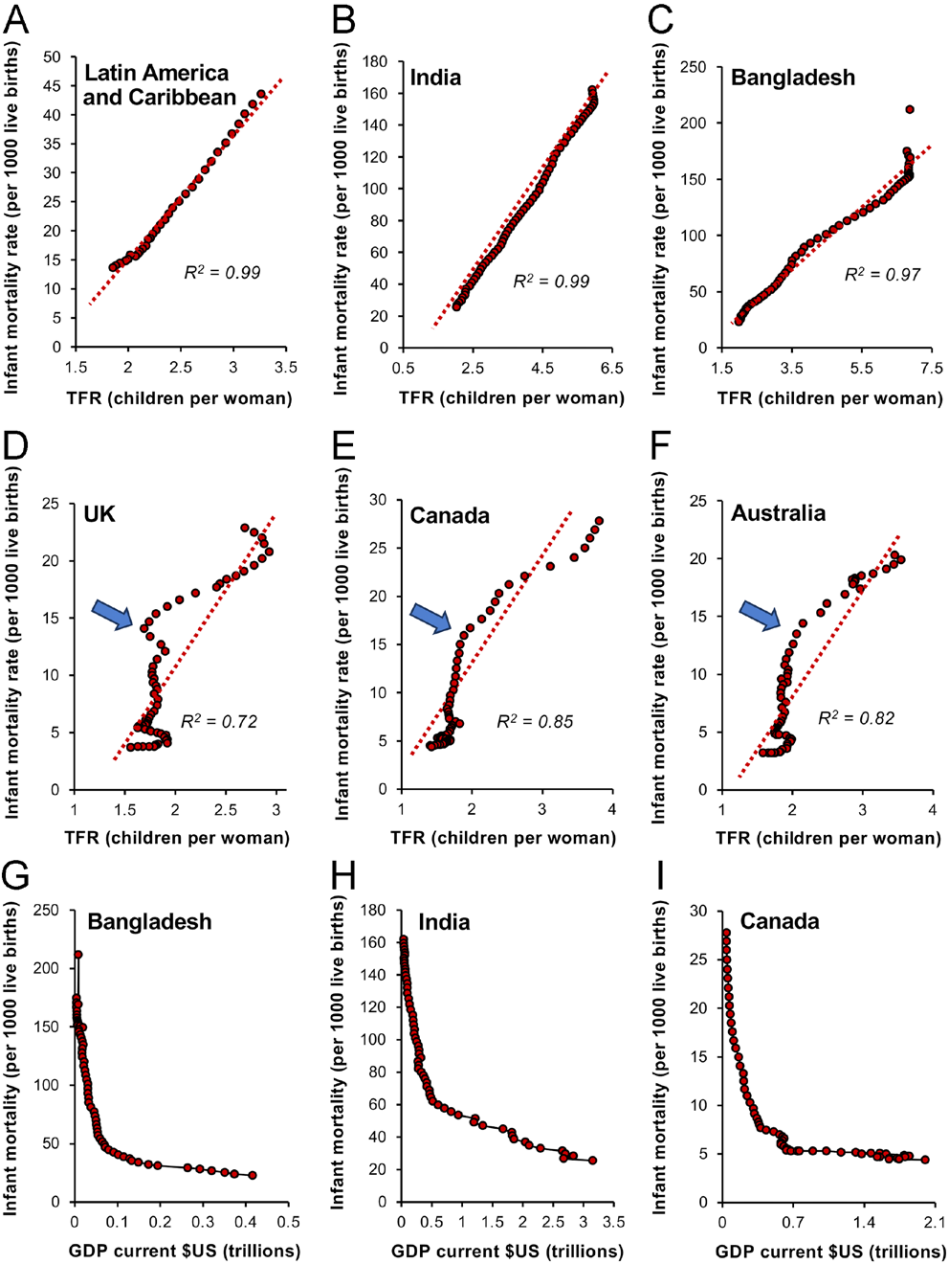
Changes in infant mortality and prosperity as important drivers of the fertility decline seen during the demographic transition. Linear correlations exist between the incidence of infant mortality and TFR; the lower the mortality, the lower the TFR. Exemplars are (A) Latin America and the Caribbean, (B) India, and (C) Bangladesh. This is even true in countries like (D) the UK, (E) Canada, and (F) Australia where the demographic transition was already well established by 1960. In these countries, the rate of TFR decline began to lessen as sub-replacement values were reached and infant mortality had declined to around 15 deaths per 1000 live births (arrowed). The decline in infant mortality is, in turn, highly dependent on an increase in GDP. Just a small increase in the latter precipitates a marked reduction in mortality rates as exemplified by (H) Bangladesh, (I) India, and (J) Canada. Source: World Bank Open data (https://data.worldbank.org/indicator/SP.DYN.IMRT.IN and https://data.worldbank.org/indicator/NY.GDP.MKTP.CD).

Extremely close correlations are observed between infant mortality and TFR decline both globally (*R^2^* = 0.94; *P* < 0.001) and in particular geographical areas, as illustrated in Fig. 4 by reference to Latin America and the Caribbean (*R^2^* = 0.99; *P* < 0.001; Fig. 4A), India (*R^2^* = 0.99; *P* < 0.001; Fig. 4B), and Bangladesh (*R^2^* = 0.97; *P* < 0.001; Fig. 4C). Even countries that have undergone the demographic transition some time ago, like the UK (*R^2^* = 0.72; *P* < 0.001; Fig. 4D), Canada (*R^2^* = 0.85; *P* < 0.001; Fig. 4E), and Australia (*R^2^* = 0.82; *P* < 0.001; Fig. 4F), show correlated declines in infant mortality and TFR over the past 62 years. However, in these cases, the relationship is not strictly linear, because TFR values change more slowly once they enter the sub-replacement zone and infant mortality rates have descended below the 15 per thousand live births threshold (Fig. 4D–F, arrowed). Overall, there is a general consensus that a reduction in infant mortality is the *sine qua non* prerequisite for fertility decline (Yamada 1985, Hondroyiannis & Papapetrou 2002).

### Prosperity

A reduction in infant mortality is itself dependent on an increase in the quality of primary healthcare that is generally related to the socioeconomic status of a given country. Just a small increase in national prosperity leads to a rapid fall in infant mortality and this relationship is evident across the socio-economic spectrum from Bangladesh to India and Canada (Fig. 4H–J). In societies that are secure and prosperous, parents no longer have to engineer large families as a buffer against the ravages of disease and starvation on their infant offspring. In the near-certain knowledge that their children will survive, they can now invest more of their precious resources in their children’s development in a quality–quantity trade-off – and family size declines (Lawson & Borgerhoff Mulder 2016).

### Urbanization

**Figure 5:**
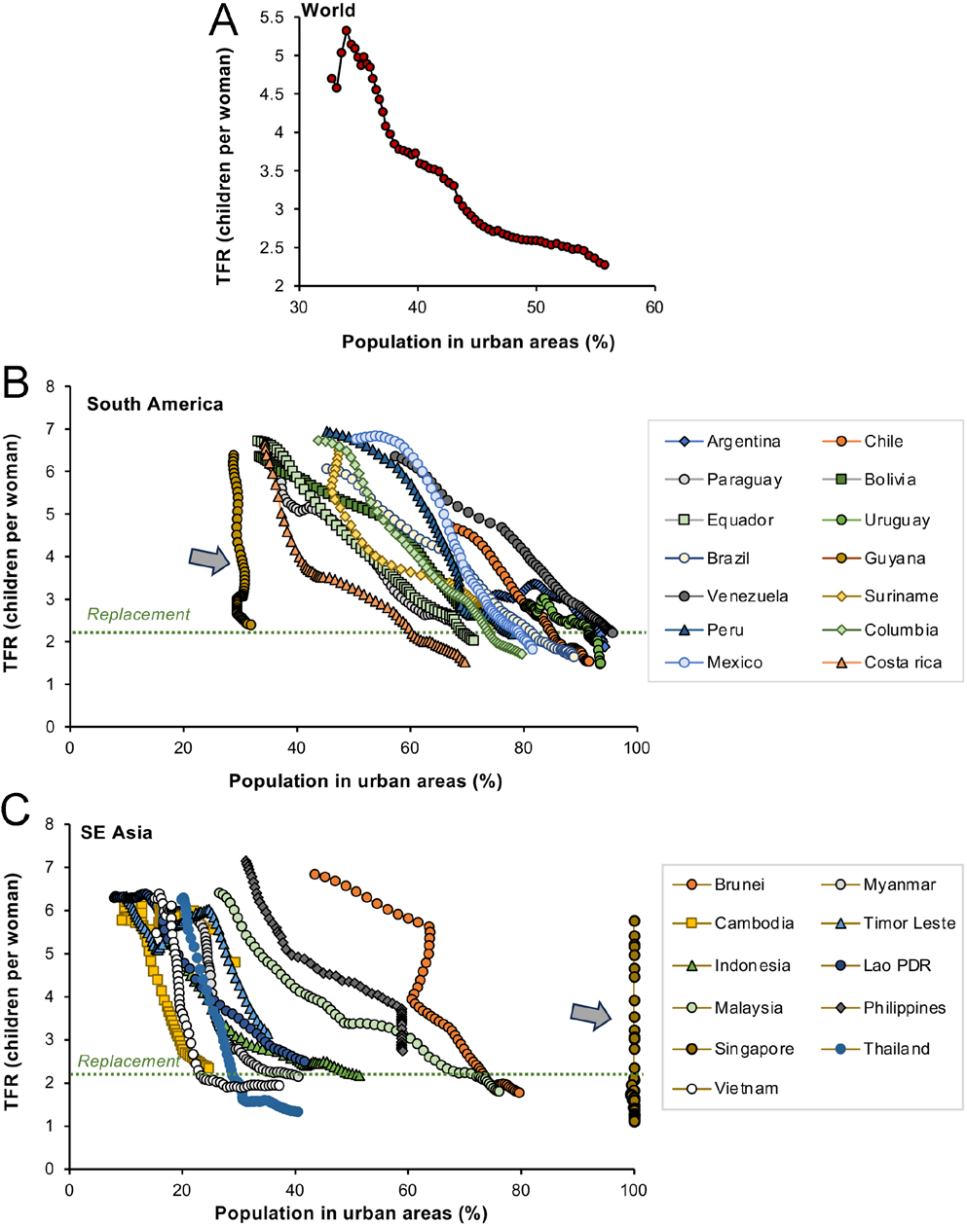
Inverse relationship between the proportion of the population living in urban areas and TFR. (A) Worldwide trend (B) South America, and (C) SE Asia. Source: World Bank Open data (https://data.worldbank.org/indicator/SP.DYN.TFRT.IN and https://data.worldbank.org/indicator/SP.URB.TOTL.IN.ZS)

The desire to invest more resources in the development of a smaller number of children encourages the movement of labour away from the land to large cities and larger conurbations, where the opportunities for work and revenue generation are increased. This movement of labour away from the country and into the city has been ongoing since the Industrial revolution and clearly correlates with economic prosperity and the global reduction in TFR (Fig. 5A). This relationship between urbanization and fertility is complex however (Martine *et al.* 2013) as illustrated in Fig. 5 in relation to South America (Fig. 5B) and SE Asia (Fig. 5C). It is clear that in both of these geographical regions, there is a general negative relationship between fertility rates and the proportion of the population living in urban environments. However, this relationship is not that precise. Thus, for most countries in South America, TFR values declined below the replacement threshold of 2.1 when more than 60% of the population was living in urban areas. SE Asia, on the other hand, is a less urban part of the world and the descent to sub-replacement levels of fertility was generally achieved when a much lower percentage of the population was living in urban areas. In Vietnam, for example, this threshold was crossed when only 24% of the population was urbanized (Fig. 5C). In addition, there are clear outliers in these data sets. Guyana has seen its fertility rates plummet with no increase in the levels of urbanization (Fig. 5B arrowed), while Singapore managed a very high TFR value (5.76) when 100% of its population were living in the city (Fig. 5C arrowed). Clearly, the global decline in TFR is more complex than a simple increase in the levels of urbanization. Nevertheless, urban environments clearly discourage large families for a variety of reasons. Living space in the city is both more limited and more expensive than in rural areas. The cost of educating and rearing children is also higher in urban than rural environments, and large cities are characterized by the presence of myriad social distractions and professional opportunities that may further move the focus away from procreation as the primary purpose of life.

### Female education

One of the most powerful influences on fertility in modern urbanized society is female education; the greater the proportion of women experiencing secondary education, the lower the fertility rate (Aitken 2022*a*,*b*). One of the major mechanisms by which female education limits fertility is through a delay in initiating a family (Bongaarts 2003, Balasch & Gratacós 2012). Delayed childbearing is a particular problem for our species because women rapidly lose their fertility after the age of 36. This decline in fertility is largely due to a loss of developmental potential on the part of the oocyte due to high levels of oxidative stress and the onset of aneuploidy in a majority of eggs (Mikwar *et al.* 2020, Jaswa *et al.* 2021, Aitken *et al.* 2022, Kordowitzki *et al.* 2024). Unfortunately, such age-dependent infertility cannot be rescued by the ART industry and, as a result, live birth rates following assisted conception show the same decline with maternal age as we see with natural conceptions (Padilla & Garcia 1989). This is not widely appreciated, with the result that many ageing women in modern society have a futile engagement with the IVF industry and, as a consequence, have families that are smaller than they would wish (Weston *et al.* 2004).

### Contraception

Educated women are also better informed about contraception, the availability of which is increased in urban compared with rural areas (Ross 2021). Globally, there is a strong negative correlation between contraceptive use and TFR (Götmark & Andersson 2020). However, the nature of this relationship is complex and shaped by two conflicting discourses. First of all, state governments, particularly in developing countries, may coerce individuals into adopting contraceptive practices in order to meet formal population targets (Senderowicz 2019). We see evidence of this in India, sub-Saharan Africa, and China where family planning services have been used as an instrument of government to rein in high rates of population growth (Gietal-Basten 2017, Senderowicz 2019). In such situations, the link between TFR and contraceptive use is clearly causative. However, this cannot be universally the case because there are many examples of countries where TFR rates have declined dramatically without large-scale support from the family planning industry. In Albania, for example, fertility rates decreased dramatically between 1960 and 1990 even though contraception was not legalized in this country until 1992. Strongly Catholic countries like Mexico, Brazil, Paraguay, and Philippines have also experienced dramatic declines in TFR since the 1960s, despite the lack of any official support for ‘unnatural’ methods of family planning over this period of time (Tsui, 2001). In Japan, the oral contraceptive pill was first made legally available in 1999 but had very little impact on Japanese TFR, which had already fallen dramatically by that point (Aitken 2022*a*). In general, the global decrease in TFR since the 1960s has not been induced by contraception – it has been enabled by it. Females in modern, advanced societies have the freedom and autonomy to make their own decisions about contraceptive use. In such situations, contraception enables women to exercise their individual choice to delay childbearing until a time that is appropriate in terms of their individual life journey. The capacity to make such reproductive decisions is fuelled by the autonomy, education, and urbanization associated with modern advanced societies and facilitated by the availability of a range of contraceptive methods to suite each woman’s individual needs.

## Social determinants of low fertility

The extent to which educated women wish to limit their family size is not just a response to the pressures of developing a professional career or resource allocation in the quality–quantity trade-off. It may also be dependent on their perceived concept of an ideal family size (Lutz 2020). The latter is often driven by an individual’s lived experience. Thus, if they come from a small one- to two-child family, their desire is often to recapitulate that experience in their own families. This relationship between family of origin and ideal family size is widespread across different countries, particularly in those undergoing the demographic transition (Booth & Kee 2009, Buhr *et al.* 2018, Beaujouan & Solaz 2019, Karhunen *et al.* 2023). Decisions over family size are also reinforced by the desire to conform to societal norms reflected in the local community or advocated by social media (Lois & Arránz Becker 2014, Barrett *et al.* 2020). The importance of such social influences means that as global fertility rates decline, so our notion of the ideal family size also becomes revised in a downwards direction. It is a self-perpetuating spiral of social contagion. In China, the one-child-family policy has created a generation of young adults for whom a small family is not only their lived experience but also their ideal (Chen *et al.* 2023).

The progressive spread of voluntary childlessness as a positive lifestyle choice, and the propagation of this concept via the internet will also make its own contribution to perpetuating the low fertility experienced by socio-economically advanced nations as they navigate the demographic transition (Koropeckyj-Cox & Pendell 2007, Noordhuizen *et al.* 2010, Shapiro 2014, Thornley 2022, Hwang 2023, Miettinen & Szalma 2014). Indeed, the concept of a childfree existence has become so socially seductive that some countries such as Russia have sought to ban the propagation of this ‘dangerous’ ideology, introducing a bill to counter the spread of this ‘destructive social behaviour based on the voluntary refusal to have children, which runs counter to traditional family values and state policy of the Russian Federation’ (The Times 2022).

## The primacy of the individual

A key cultural element in the journey to sub-replacement levels of fertility has been the rise of individualism and a re-focusing of life’s purpose away from the traditional nuclear family towards self-fulfilment and the realization of potential. Historically, this journey began in the late 18th century with the Enlightenment. Pioneered by key figures such as Newton, Locke, and Kant, the Enlightenment was a philosophical movement that promoted the notion that our life course should not be determined by State or Religion but guided by reason. It is through reason that we gain an understanding of the universe and the ability to improve our own condition (Peters 2019). Ultimately the ideas promulgated by the Enlightenment have had a major impact on the human condition and our demographic future. They have provided us with the cultural foundations of a free, modern society, captured by the ‘developmental idealism’ cultural model and characterized by education-for-all, good health, freedom, autonomy, gender equality, demographic governance, and small families (Thornton 2001, Thornton *et al.* 2015). The ideas seeded by the Enlightenment and enabled by the prosperity provided by the Industrial Revolution have simultaneously led to a progressive change in our vision of life’s purpose. We have seen a gradual shift away from formal religion or procreation as the guiding principles of existence. The autonomy associated with modern society has provided its citizens with the freedom to determine the place of family in their life trajectories and, for all the reasons provided previously, that choice frequently falls on the side of low fertility (Vitali *et al.* 2009).

## Migration

**Figure 6:**
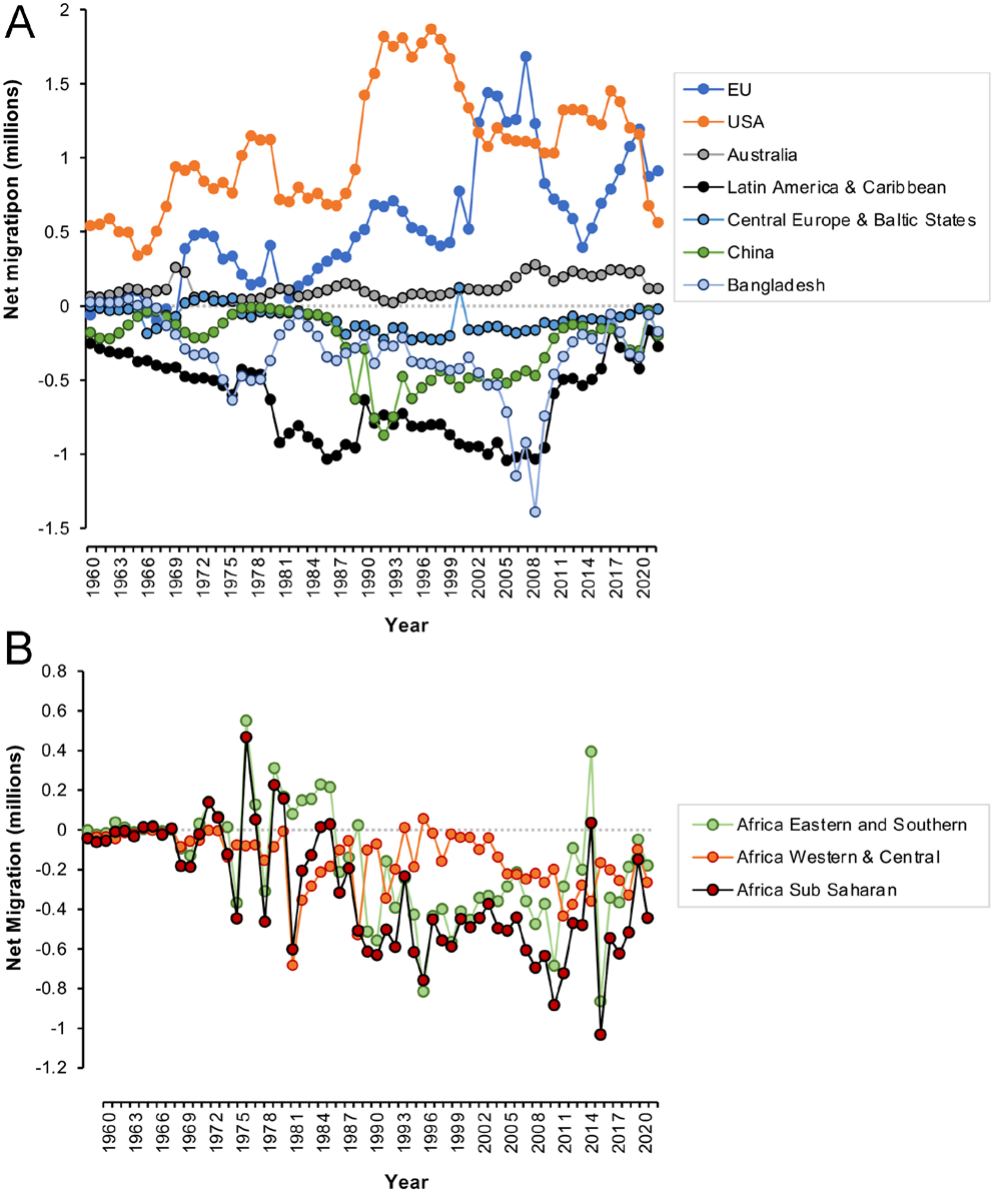
Patterns of migration as a key determinant of population dynamics. (A) Net recipients of immigrants are the EU, the USA, and Australia, while significant emigration has been observed from area such as Latin America and the Caribbean, Central Europe, and the Baltic States, as well as China and Bangladesh. Although the annual migration figures are very variable, the evidence suggests a recent return to low levels of emigration from the ‘supplier’ nations to reach levels similar to those observed in 1960. (B) Related data for different regions of Africa, which also shows early signs of reversing the net emigration that has dominated the population profiles of these countries for the past half century. Source World Bank Open Data (https://data.worldbank.org/indicator/SM.POP.NETM).

Another unexpected consequence of the autonomy fostered by the Enlightenment is the importance of migration in determining population dynamics at a national level. Individuals no longer feel constrained by the limitations of the country into which they were born. In the 21st century, the personal autonomy conferred by modern society means that individuals feel they have a right to migrate to more prosperous, secure, liberal regions of the world to facilitate the realization of their potential and enhance their quality of life. The rapidly declining populations we are seeing in the Balkans, Central Europe, and Baltic States (Fig. 3) are not just the result of low fertility levels but are also facilitated by high rates of emigration (Finnsdottir, 2019). Figure 6 shows some of the net beneficiaries and losers in the migration game. Global regions such as the USA, Australia, and the EU are net recipients of immigrants and use this as a device to maintain the momentum of their economies and paper over the cracks of their intrinsic infertility (Fig. 6A). Net providers of migrants are areas such as Latin America and the Caribbean, Central Europe and the Baltic States, China, the Indian subcontinent, and most parts of Africa (Fig. 6A and B). The yearly net migration figures for these donor nations are extremely variable, reflecting the vulnerability of migration to the impact of myriad factors including global pandemics, environmental change, economic pressure, availability of work, skill shortages, and political expedience. Notwithstanding this variability, it is evident that over the past 20 years, the emigration door has been slowly closing for some of the major supplier nations including China, Latin America, and the Caribbean as well as Bangladesh (Fig. 6A). Migration is clearly an effective mechanism for some prosperous nations to counter their declining fertility, but it is too susceptible to interference from multiple sources (not least the will of supplier nations) to be anything other than a short-term measure.

## Age structure and fertility rate

One of the long-term consequences of a reduction in fertility rate is a change in the age structure of the population. In stages 1 and 2 of the demographic transition, societies are characterized by a triangular age profile with a broad base and narrow apex. Under these circumstances, a largely youthful work force is comfortably able to provide the resources needed to support the limited number of individuals who make it through to the ranks of the elderly (Box 1). However, following the demographic transition, we are faced with a very different state of affairs, because the age profile of the population becomes more and more inverted. In most modern advanced economies, you have a shrinking youthful workforce working extremely hard to generate the resources needed to support a growing throng of aged citizens (Box 1). This not only places the aged care system under immense pressure (Chen *et al.* 2020, Feng *et al.* 2020) but also impacts the stretched workforce itself. The modern workplace is associated with high levels of psychological stress culminating in professional burn-out and obsessive workaholic behaviour patterns (Weber & Jaekel-Reinhard 2000, Andreassen 2014, Barsties *et al.* 2023). If the young adult work force is psychologically stressed, financially penalized, time-poor, burnt out, and unable to access affordable accommodation, is it any wonder that their desire to have a family suffers? (Sheiner *et al.* 2002, Palomba *et al.* 2018).

## Long-term consequences of low TFR

Once the above collection of socio-economic factors has driven societies to extremely low levels of fertility, what happens next? At this stage of development, societies are said to have engaged the second transition characterized by sustained ‘lowest-low’ fertility levels (TFR < 1.3), cultural individualism, postponed marriage and parenthood, a disconnection between marriage and procreation, high rates of divorce, more co-habitation, frequent contraceptive use, a rising incidence of voluntary childlessness, disengagement from civic and community-orientated networks, denial of authority, greater gender equity, increased female economic autonomy, fluid life course arrangements and a shifted age structure in favour of the elderly (Lesthaeghe 2014) – all features of modern society that we can instantly recognize. Whether the extremely low fertility associated with the second transition is indicative of a permanent societal change or whether we shall revert back to higher levels of fertility should circumstances so dictate is a question that has been much debated by social demographers with contrasting conclusions (Lutz *et al.* 2006, Goldstein *et al.* 2009).

Many authors have sensed the green shoots of fertility revival in the past, only to be frustrated in the long run, by the stubborn refusal of fertility rates to climb back above the replacement threshold (Morgan 2003, Goldstein *et al.* 2009, Myrskylä *et al.* 2009). Indeed, it is difficult to imagine why there would be an uplift in TFR if the socio-economic (female education, urbanization, developmental idealism, increased autonomy, financial hardship, etc.) and health-orientated (low infant mortality, delayed childbearing, and contraceptive availability) drivers for low fertility have not changed.

Nevertheless, if the only causes of low fertility are socio-economic and cultural in nature, then, theoretically, there is no reason why we could not reverse the fertility decline should the desire be there to do so, and Governments adjust their policy settings appropriately. However, if low fertility levels persist for a prolonged period of time, then this choice may be taken away from us by a combination pollution, evolution, and, paradoxically, the assisted conception industry.

## Natural selection

In modern society, when infant mortality rates are low, family sizes are small and ART is available to address any traces of infertility (World Health Organization 2023), selection pressure for high fecundity genes is removed. In a modern, developed society a given couple may possess a combined high fertility genotype capable of generating a large family but decide to remain childfree. Conversely a couple may possess a combined low fertility genotype incompatible with natural conception but, with the aid of ART, decide to have a family of three. This lack of evolutionary selection pressure will ultimately result in increased variation within the cohort of genes supporting reproduction and, since most non-neutral mutations are deleterious, our fecundity (i.e. our fundamental capacity to reproduce) may ultimately suffer as a result. We are already seeing an increase in such genetic variance within the human population (Bolund *et al.* 2015) and are becoming increasingly aware of the significant role that genetics and epigenetics are playing in the aetiology of human fertility (Gunes & Esteves 2021, Sang *et al.* 2023).

## Environmental pollution

The future of human fertility is also a reflection of the polluted state of the environments associated with many modern industrialized societies. Incontrovertible evidence for an impact of environmental toxicants on the reproductive system can be found in the increased incidence of testicular cancer, which rises exponentially in developed countries as their TFR values fall below replacement levels (Aitken 2022*a*, *b*). This change is thought to reflect the direct impact of environmental toxicants on the development of the male reproductive system during a critical stage of foetal development (Sharpe & Skakkebaek 2008, Skakkebaek *et al.* 2022). According to the testicular dysgenesis model, environmental toxicants associated with modern society are not only increasing rates of testicular cancer but also inducing other developmental defects in the male including cryptorchidism and hypospadias (Skakkebaek 2003). In addition, the same pollutants are thought to be responsible for a worldwide decline in sperm counts which, if the present rate of decline continues unabated, will ultimately have an impact on the long-term fecundity of our species (Levine *et al.* 2023). Not surprisingly, environmental toxicants also impact the female reproductive system (Dutta *et al.* 2022, Go *et al.*, 2023) and are thought to play a direct role in the causation of infertility across both sexes (Canipari *et al.* 2020, Erenpreisa *et al.* 2023).

One of the mechanisms by which environmental toxicants might increase the incidence of human infertility is by triggering the creation of *de novo* genetic and epigenetic mutations in the germ line that impact our capacity to procreate. Since *de novo* mutations are predominantly associated with the male germ line, it has been suggested that the induction of DNA damage in spermatozoa is a critical element of this mutagenic process. According to this ‘post-meiotic oocyte collusion’ model, environmental toxicants such as bisphenol A or phthalate esters induce high levels of DNA damage in the spermatozoa, which are then converted into mutations/epimutations as a result of aberrant or deficient DNA repair in the fertilized egg (Rubes *et al.* 2005, Somers 2011, Aitken 2022*c*). Since a significant proportion of the genome is involved in regulating reproduction (Mouse Genome Sequencing Consortium *et al.* 2002), it would not be surprising if many of these random genetic mutations affected the fecundity of our species.

## Assisted reproductive technology

The mutagenic potential of 21st-century environments (Naidu *et al.* 2021) combined with the lack of selection pressure on our reproductive capacity will therefore encourage the generation of *de novo* mutations affecting human fertility. Furthermore, the assisted conception industry may inadvertently promote this process, by helping to retain poor fertility genotypes/epigenotypes within the population. In particular, techniques such as ICSI, which permit conceptions *in vitro* that would have prevented *in vivo*, may play a significant role in the spread of mutations that are damaging to human fertility. For example, AZFc mutations on the Y-chromosome are known to result in severe male infertility but can be propagated by ICSI, generating infertility in any male offspring conceived in this manner (Page *et al.* 1999). Similarly, a wide range of mutations that affect the morphology and function of the sperm tail including Kartagener syndrome (Dávila Garza & Patrizio 2013) and multiple morphological anomalies of the sperm flagellum (Ferreux *et al.* 2021, Shao *et al.* 2023, Song *et al.* 2023) can be treated with ICSI, potentially impacting the fertility of any male offspring. Preliminary data indicating that median sperm concentration, total sperm count, and total motile sperm count are significantly lower in ICSI conceived males than their spontaneously conceived counterparts is also suggestive that ART can enhance the vertical transmission of poor fertility genotypes (Belva *et al.* 2016). Such relationships are of little consequence while ART remains a cottage industry responsible for less than 1% of births worldwide. However, this technology is now contributing to more than 5% of annual births in Australia and in Scandinavian countries like Denmark, this figure is as high as 10%. This may not be a concern at the present time, however, while the vector of ART uptake remains sharply upwards, we should be aware that this technology, designed to enhance human fertility and responsible for millions of births worldwide, could, when practiced at scale, inadvertently compromise this process and create a dependence on ART that will challenge both healthcare providers and patients alike.

## Conclusion

The march of human progress is leaving a trail of infertility in its wake. All over the world, fertility rates are in retreat and in some countries, we are already seeing a significant impact on population numbers. Migration is providing temporary relief to some nations, but this can only ever be a relatively short-term measure. Ultimately, all nation-states will have to come to terms with their shrinking, ageing population profiles. These trends could readily be perceived in a positive, Neo-Malthusian, light since a reduction in population pressure would surely yield environmental dividends relevant to a plethora of difficulties facing our species including climate change, the collapse of biodiversity, the loss of precious natural resources, the spread of disease, widespread pollution, etc. Conversely, a rapid decline in population numbers could also have negative economic, social, and geopolitical consequences if we are not prepared for such a change.

In the end, low fertility should not be judged as ‘good’ or ‘bad’. Rather, we should aspire to be managers of our population dynamics rather than victims. Achieving this end will require a detailed knowledge of the mechanisms responsible for the global decline in fertility rates so that the necessary range of policy settings and healthcare strategies can be introduced to control population numbers in whatever direction is deemed appropriate. Policy changes addressing the distribution of taxation load, the provision of adequate parental leave schemes and childcare facilities, access to affordable accommodation, changes in employers’ attitudes towards maternity/paternity leave, are all part of the short-term measures required to help couples have children if they so desire. The prioritization of reproductive toxicology and awareness on the part of environmental protection agencies of the risks posed by environmental pollutants to human fertility, would also be helpful in the longer term (Aitken 2022*a*,*b*). It would also help if we spent more time trying to understand the aetiology of human infertility instead of treating every case with ART. Above all, we must appreciate the fragility of human fertility and do everything we can to educate and support young couples in both making and achieving their reproductive choices.

## Declaration of interest

RJ Aitken is an associate editor of *Reproduction* and was not involved in the review or editorial process for this paper, on which he is listed as an author. The author declares that there is no conflict of interest that could be perceived as prejudicing the impartiality of this point of view article.

## Funding

This work did not receive any specific grant from any funding agency in the public, commercial, or not-for-profit sector.
